# CARD9 Forms an Alternative CBM Complex in Richter Syndrome

**DOI:** 10.3390/cancers14030531

**Published:** 2022-01-21

**Authors:** Julia Maier, André Lechel, Ralf Marienfeld, Thomas F. E. Barth, Peter Möller, Kevin Mellert

**Affiliations:** 1 Institute of Pathology, University Hospital Ulm, 89081 Ulm, Germany; julia.maier@uniklinik-ulm.de (J.M.); ralf.marienfeld@uniklinik-ulm.de (R.M.); thomas.barth@uniklinik-ulm.de (T.F.E.B.); kevin.mellert@uni-ulm.de (K.M.); 2 Department of Internal Medicine I, University of Ulm, 89081 Ulm, Germany; andre.lechel@uni-ulm.de

**Keywords:** Richter syndrome, chronic lymphocytic leukemia, NF-κB

## Abstract

**Simple Summary:**

The transformation process of chronic lymphocytic leukemia into an aggressive lymphoma, called Richter syndrome (RS), is incompletely understood, and therapeutic options are limited. Here, we report *CARD9* to be expressed in a subset of RS tissue specimen and in the first and only available RS cell line, U-RT1. In U-RT1, CARD9 attaches to BCL10 and MALT1, and knockdown of CARD9 leads to a significant reduction in cell viability. We hypothesized that CARD9 plays an oncogenic role in RS through the activation of NF-κB signaling. Our findings may help to extend the current knowledge about the pathogenesis of RS and promote the development of targeted therapies for this aggressive disease.

**Abstract:**

Richter syndrome (RS) is defined as the transformation of chronic lymphocytic leukemia (CLL) into an aggressive lymphoma, mostly diffuse large B-cell lymphoma (DLBCL). Despite intensive therapy, patients with RS have an unfavorable clinical outcome. The detailed pathobiology of Richter transformation still needs to be elucidated. Here, we report high mRNA and protein levels of *CARD9* in the RS cell line U-RT1. Co-immunoprecipitation revealed the assembly of a CBM complex using CARD9 instead of CARD11. CARD9 is known to be an activator of NF-кB signaling in myeloid cells. U-RT1 Western blot analyses showed phosphorylation of IκB as well as IKK, indicating a constitutively active canonical NF-кB pathway. This was further supported by the significant reduction in cell viability and CYLD cleavage products after CARD9 siRNA knockdown. We also showed immunostaining for CARD9 in 53% of cases analyzed in a series of RS tissue specimens, whereas other lymphomas rarely show *CARD9* expression. This is the first report on ectopic expression and function of CARD9 in an aggressive B-cell lymphoma. Our findings suggest that CARD9 may contribute to the pathogenesis of RS.

## 1. Introduction

Chronic lymphocytic leukemia is an indolent B-cell neoplasm that, in 5–10% of cases, will undergo malignant transformation into an aggressive B-cell lymphoma, most commonly a diffuse large B-cell lymphoma [1,2], termed Richter syndrome (RS) [3]. Diagnosis requires a histologic shift to DLBCL that must be distinguished from an “acceleration” of CLL with expansion of proliferation centers [4]. Besides the DLBCL variant, a classical Hodgkin lymphoma (cHL) variant of RS can occur, but unusual variants such as plasmablastic lymphoma and high-grade T-cell lymphoma have also been described [5,6]. RS DLBCL cells invariably express CD19, but tend to lose the expression of CD5 and CD23, leading to a morphologic and immune phenotype different from the underlying CLL [7]. Using the Hans algorithm [8], 90–95% of RS DLBCL are identified as the more aggressive activated B-cell subtype (ABC DLBCL) [1,7,9]. Despite intensive therapy, patients with RS have a poor clinical outcome, with a median survival of 1–2 years from diagnosis [10,11]. A clonal relationship to the preceding CLL has been shown to be an important unfavorable prognostic factor [9].

Advances in the field have unraveled multiple clinical and biological risk factors for the development of RS, including an advanced stage of CLL [12,13], nonmutated *IGHV* [9,12], absence of del13q14, and mutated *NOTCH1* [14,15], as well as inactivation of *TP53* or del17p13 [2,13]. Although specific types of RS differ in their underlying aberration profile [9], 90% of RS cases show aberrations in *TP53*, *NOTCH1*, *MYC*, or *CDKN2A*, thus affecting more general regulators of apoptosis and proliferation [16,17]. Recently, Chakraborty et al. showed that biallelic loss of TP53/CDKN2A/2B induced BCR-dependent proliferation of CLL cells and transformation into an aggressive RS-like phenotype in vivo and in vitro [18]. In addition, AKT activation was identified as an inductor of CLL transformation toward aggressive lymphoma via Notch1 signaling in a murine CLL model by Kohlhaas et al. [19]. Importantly, genetic lesions that characterize de novo DLBCL, such as the *EZH2* or *CREBPP* mutations commonly found in the germinal center B-cell-like (GCB) subtype of DLBCL or the perturbations of the B-cell signaling pathway seen in the ABC DLBCL subtype, do not seem to be prevalent in RS DLBCL. These data indicate that RS is a lymphoma entity distinct from de novo DLBCL [9,17].

The majority of de novo ABC DLBCL show a constitutively active NF-кB pathway as a potent antiapoptotic program [20]. In approximately 10% of ABC DLBCL, gain of *CARD11* function mutations plays a pivotal role in activating NF-кB and driving lymphomagenesis [21]. In non-neoplastic B cells, BCR signaling leads to PKCβ activation, which then phosphorylates CARD11 and converts it to an active state [22]. Subsequent assembly of the CBM complex composed of active CARD11, MALT1 paracaspase, and BCL10 activates IкB kinase (IKK), which then phosphorylates the inhibitor of NF-кB (IкB). Phosphorylation of IкB results in degradation of the inhibitor, release of the NF-кB proteins into the nucleus, and expression of NF-кB target genes [20]. Induction of the NF-кB pathway is crucial for ABC DLBCL, since silencing of any component of the CBM complex or inhibition of IKK is toxic to ABC DLBCL [21,23].

In view of the unfavorable prognosis and limited therapeutic options, further investigations to elucidate the pathobiology of RS are needed. In this study, we used the recently established first and only available RS cell line, U-RT1 [24], as a new model system for RS in order to explore possible pathogenetic mechanisms.

## 2. Materials and Methods

### 2.1. Cell Culture

Cell culture reagents were obtained from Lonza (Basel, Switzerland), and fetal calf serum from Seromed/Biochrom (Berlin, Germany). All cell lines were grown in IMDM/RPMI (4:1) supplemented with 10% fetal calf serum, glutamine, and 100 U/mL L-penicillin/streptomycin at 37 °C and 5% CO_2_. For the production of cell pellets, cells were precipitated with pure alcohol, subsequently fixed in 5% formalin solution, and embedded in paraffin. 

### 2.2. Immunochemistry and Immunofluorescence

Immunochemistry was performed on formalin-fixed, paraffin-embedded (FFPE) tissue and cell pellets using the avidin–biotin complex method with the AP/RED Detection System (K5005, Dako, Hamburg, Germany). The following antibodies were used: rabbit anti-CARD9, 1:100 (C7862, Sigma-Aldrich, St. Louis, MO, USA); mouse CD20, 1:500 (L26, Dako); and mouse anti-KI67, 1:200 (MIB1, Dako). For immunofluorescence double staining, Cy3-conjugated goat anti-mouse IgG, 1:400 (Jackson Immunoresearch, Westgrove, PA, USA) was used to detect the CD20 antibody; and biotin-conjugated pig anti-rabbit IgG, 1:200 (DAKO) followed by signal amplification with the Alexa Fluor-488 kit, 1:1600 (Thermo Fisher, Waltham, MA, USA) detected the anti-CARD9 antibody. Images were processed using ISIS3 software (Metasystems, Heidelberg, Germany).

### 2.3. Microarray Gene Expression Profiling

Total RNA was extracted from cell lines with the RNeasy Mini-Kit (Qiagen, Hilden, Germany) following the manufacturer’s instructions. Gene expression analysis was performed using the Human Gene Expression 4 × 44 K Microarray Kit (Design ID 014850, Agilent Technologies, Santa Clara, CA, USA). A total of 200 ng RNA per sample was used and processed according to the standard protocol (Agilent Technologies, Santa Clara, CA, USA). Analyses were performed in triplicate. Signals were captured by a Microarray Scanner (G2565, Agilent Technologies), and data were analyzed with Genespring 12.1 (Agilent Technologies). Expression data of U-RT1 were compared with published data on the HBL-1, OCI-Ly3, OCI-Ly7, and SUDHL6 cell lines with the following signatures: Array Express E-MTAB-973 and GEO Accession Number GSM466597.

### 2.4. Quantitative Real-Time PCR

The mRNA expression levels were determined by real-time reverse transcriptase (RT)-PCR. β-Actin and GAPDH served as internal controls. A NanoDrop 2000 spectrophotometer (Thermo Fisher) was used to measure RNA quality and quantity. The SuperScript II system (Invitrogen, Carlsbad, CA, USA) was used to synthesize single-strand cDNA from oligo-dT-primers according to the manufacturer’s instructions, and qPCR was performed with the QuantiTect SYBR Green PCR Kit (Qiagen) using a LightCycler Rotor Gene Q (Qiagen). For all target genes, exon–exon boundary primers were designed to minimize amplification of traces of DNA. Primer sequences are documented in Supplementary Table S1.

The PCR reaction was performed as follows: the initial denaturation step was at 95 °C for 5 min, followed by 45 cycles at 95 °C for 10 s and 60 °C (*CARD9*, CARD11, MALT1, and BC*L10*) or 51 °C (β-*Actin*) for 20 s. A melting curve analysis was performed for each experiment, and PCR products were analyzed by agarose gel electrophoresis to confirm specificity. All experiments were performed in triplicate. Quantification was performed using the established ΔΔCT method.

### 2.5. Gene Sequencing 

DNA was isolated from U-RT1 cells with the QiAmp DNA Mini Kit (Qiagen). Gene sequencing of *MYD88* (exon 5) and *CD79A* (exon 5 + 6) was performed according to a standard protocol for diagnostic purposes using a Multiplex PCR Kit (Qiagen, cat. no. 206143) and MiSeq-Sequencer (Illumina, San Diego, USA, cat. no. Y-410-1003). Primer sequences are documented in Supplementary Table S1. For sequencing *CARD9* (all exons) and *CARD11* (all exons), a custom-made Gene Read V2 chemistry panel (Qiagen) was applied. Target enrichment, amplicon processing, and library generation were performed as suggested by the manufacturer.

### 2.6. Clonality Testing (Heavy Chain Rearrangement) 

The rearrangement of the immunoglobulin heavy chain gene was determined by multiplex-PCR using framework region 1, 2, and 3 (FR1, FR2, and FR3)-specific primers as described by Dongen et al. (2003 Leukemia) and as previously described in Schmid et al. [24].

### 2.7. Western Blot and Co-Immunoprecipitation

Protein isolation was performed in TNT++ buffer (20 mM Tris pH 8.0, 200 mM sodium chloride, 1% Triton-X100, 1 mM DTT) containing both phosphatase and protease inhibitor cocktails (Sigma-Aldrich, St. Louis, MO, USA). Lysates were snap-frozen in liquid nitrogen for 3 min and subsequently centrifuged at 14,000 rpm for 10 min at 4 °C to remove debris. For protein quantification, the BCA Protein Assay Kit (Thermo Fisher) was employed, following the manufacturer’s instructions. Measurements were made in an EPOCH spectrophotometer (BioTek, Winooski, VT, USA) at a 562 nm wavelength.

SDS-PAGE was performed with 10–20 µg protein using Nu-PAGE 4–12% Bis-Tris protein gels (ThermoFisher). A standard semidry method using 0.2 mm nitrocellulose membranes (GE Healthcare, Chicago, IL, USA) was applied for protein transfer. Membrane blocking was done with 5% milk powder in TBS Tween 20. Primary antibodies were incubated overnight at 4 °C. After multiple washing steps and repeat blocking with 5% milk powder in TBS Tween 20, membranes were incubated for 1 h at room temperature with horseradish-peroxidase-conjugated secondary antibody. Detection was performed using the ECL substrate WesterSure (LI-COR) on a Western Blot Scanner (LI-COR Biosciences, Lincoln, OR, USA). Evaluation was done with C-DiGit Blot Scanner Software, and quantification with ImageJ (National Institutes of Health, Bethesda, MD, USA).

The following primary antibodies were used: rabbit anti-CARD9 (clone C7862, Sigma-Aldrich), rabbit anti-MALT1 (clone H-200, Santa Cruz, Dallas, TX, USA), goat anti-BCL10 (C17, Santa Cruz), mouse anti-CYLD (clone E-10, Santa Cruz), mouse anti-pIкB-α (clone B-9, Santa Cruz), rabbit anti-pIKKα/β (clone Ser176/180, Cell Signaling, Danvers, MA, USA), mouse anti-p65 (F6, Santa Cruz), and mouse anti-ß-Actin (AC-74, Sigma-Aldrich). Secondary antibodies were: goat anti-mouse IgG (Invitrogen) and goat anti-rabbit IgG (Sigma-Aldrich). 

For co-immunoprecipitation assays, 0.5–1 mg whole protein extract was mixed with 1 mg goat anti-BCL10 antibody (C17, Santa Cruz); rabbit anti-p65 antibody (AB_2793231, Active Motif, Waterloo, Belgium) served as control. After overnight incubation at 4 °C on a rotator, 10 µg protein G (Protein G Sepharose 4 Fast Flow, GE Healthcare) dissolved in 100 µL TNT++ buffer was added, and incubation was continued at 4 °C for 1 h. Immunoprecipitates were washed twice with TNT++ buffer and twice with TBS buffer before separation by SDS-PAGE and Western blot analysis as described above.

### 2.8. Inhibition with BAY11-7082 and siRNA Knockdown

To inhibit the NF-κB pathway, cells were treated for 24 h with different concentrations of the inhibitor compound BAY11-7082 (Selleckchem, Houston, TX, USA) diluted in DMSO (Sigma-Aldrich) before an MTT cell proliferation assay was performed. As a control, cells were incubated with equivalent amounts of DMSO omitting BAY11-7082. Measurements were performed in technical quintuplicates, and each test series was repeated three times.

Transfection of cell lines with siRNA was performed using the Amaxa Cell Line Nucleofector Kit V (Lonza) with program X001 according to the manufacturer’s protocol. For CARD9 siRNA knockdown, a mixture of siRNA oligonucleotides (Qiagen Flexitube Gene Solution) was used. The siRNA sequences are shown in Supplementary Table S1. Then, 24 h after siRNA transfection, cell viability was measured using an MTT proliferation assay.

The MTT proliferation assay was performed using 3-(4,5-dimethyl-2-thiazolyl)-2,5-diphenyl-2H-tetrazolium bromide (Sigma-Aldrich) dissolved in PBS. After adding 10 µL of 12 mM MTT reagent to 100 µL of cell culture and incubating for 3 h at 37 °C, the cells were lysed with 100 µL 10% SDS/0.01 M HCl. After one hour of further incubation, formazan absorbance was measured at 570 nm.

### 2.9. Statistics

For statistical analysis, the Welch two-sample *t*-test was used. A *p*-value ≤ 0.05% was considered significant. EC50 estimates for Bay11-7082 treatment were obtained from Emax models calculated using the stan_emax function from the R package rstanemax.

In MTT experiments, the paired *t*-test was used. Sample values were normalized to the individual mean of the control group, to allow a comparison across all samples at the same time. All analyses were performed using R version 4.0.0 (The R Foundation, Vienna, Austria).

### 2.10. Lymphoma FFPE Tissue Bank

We analyzed FFPE samples from 15 patients with RS, 26 patients with cHL, 10 patients with de novo DLBLC, 25 patients with CLL, and 17 patients with aggressive lymphoma transformed from indolent lymphoma. All work was performed in conformity with the Declaration of Helsinki, and the study was approved by the local ethics committee (Ulm Ethics Committee, vote 371/17). The patients gave written informed consent for the scientific use of their tumor samples.

## 3. Results

### 3.1. U-RT1 Shows High Levels of CARD9 Expression

**Figure 1:**
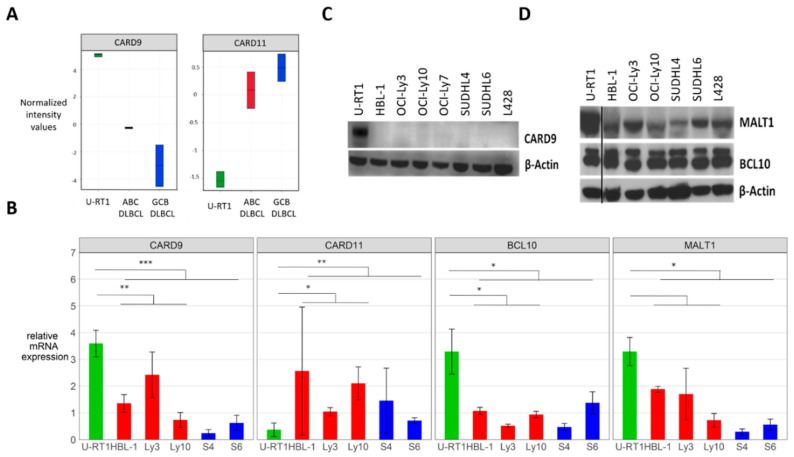
*CARD9* expression in U−RT1, L428, and de novo DLBCL. (**A**) Microarray mRNA expression profiling of *CARD9* and *CARD11* in U−RT1 (green) compared with HBL−1 and OCI−Ly3 (ABC DLBCL; red), OCI−Ly7, and SUDHL4 (GCB DLBCL; blue). (**B**) RT-qPCR expression analyses of *CARD9, CARD11, BCL10*, and *MALT1* mRNA. The figure shows values of relative mRNA expression compared with mean values of the expression of each gene in all cell lines tested. All ΔCT values were normalized to β−Actin. (**C**) Western blot analysis showed expression levels of CARD9 in U−RT1, de novo DLBCL cell lines, and L428. (**D**) Immunoblot for MALT1 and BCL10 in U-RT1, de novo DLBCL cell lines, and the cHL cell line L428. * *p* < 0.05, ** *p* < 0.01, *** *p* < 0.001. Abbreviations: Ly3 = OCI−Ly3, Ly10 = OCI−Ly10, S4 = SUDHL4, S6 = SUDHL6. All mRNA expression experiments were performed in technical and biological triplicates.

We carried out microarray expression profiling to identify genes differentially expressed in the still-unique RS cell line U-RT1 and de novo DLBCL cell lines. This revealed a high level of *CARD9* mRNA in U-RT1 compared with the ABC DLBCL cell lines HBL-1 and OCI-Ly3, as well as with the GCB DLBCL cell lines OCI-Ly7 and SUDHL4. CARD9 is known to be an important activator of NF-кB signaling in myeloid cells, forming an alternative CBM complex with MALT1 and BCL10. *CARD11*, however, was only weakly expressed in U-RT1 (Figure 1A). RT-qPCR analyses confirmed these results, also revealing higher mRNA expression of the CBM complex proteins MALT1 and BCL10 and a significantly lower *CARD11* expression in U-RT1 compared with the de novo DLBCL cell lines (Figure 1B). On the protein level, Western blotting showed CARD9 exclusively in U-RT1, whereas all the de novo DLBCL cell lines and the cHL cell line L428 lacked the CARD9 protein (Figure 1C). Immunoblotting also confirmed high levels of MALT1 in U-RT1. BCL10 protein levels in U-RT1 were comparable to the de novo DLBCL cell lines (Figure 1D).

### 3.2. Immunostaining Reveals CARD9 Expression in the U-RT1 Cell Line and in the U-RT1 Parent Lymph Node

**Figure 2:**
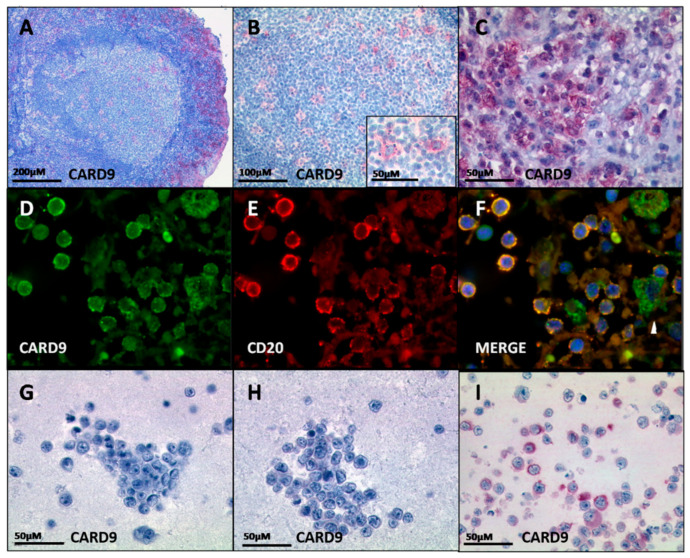
Immunostaining of CARD9. (**A**,**B**) CARD9 staining of a reactive lymph follicle. (**C**) CARD9 expression of the lymph node from which U-RT1 was established. (**D**–**F**) Double fluorescence staining of CARD9 and CD20 in the parent tissue of U-RT1. (**D**) Cytoplasmic staining for CARD9 in green. (**E**) CD20 staining in red. (**F**) Merge of (**D**,**E**); white arrowhead indicates myeloid bystander cells. (**G**–**I**) CARD9 immunostaining of DLBCL cell lines HBL-1 (**G**) and SUDHL4 (**H**), and the RS cell line U-RT1 (**I**).

*CARD9* is predominantly expressed in myeloid cells [25,26,27]. Accordingly, immunostaining of reactive lymph nodes revealed CARD9 in macrophages and dendritic cells, but not in lymphoid cells (Figure 2A,B). In contrast, the parent RS DLBCL from which U-RT1 was established contained sheets of CARD9-positive lymphoma cells (Figure 2C). Double immunofluorescence staining of CARD9 and CD20 (Figure 2D–F) demonstrated a colocalization of these two antigens, confirming the high prevalence of CARD9-positive neoplastic B cells.

We also tested the de novo DLBCL cell lines HBL-1 and SUDHL4 for CARD9 protein levels using immunostaining (Figure 2G,H). In contrast to U-RT1 (Figure 2I) these cell lines showed no immunoreactivity for CARD9. Taken together, these results confirmed the exceptional expression of *CARD9* in U-RT1.

### 3.3. CARD9 Is Expressed in a Subset of Richter Syndrome Cases

**Figure 3:**
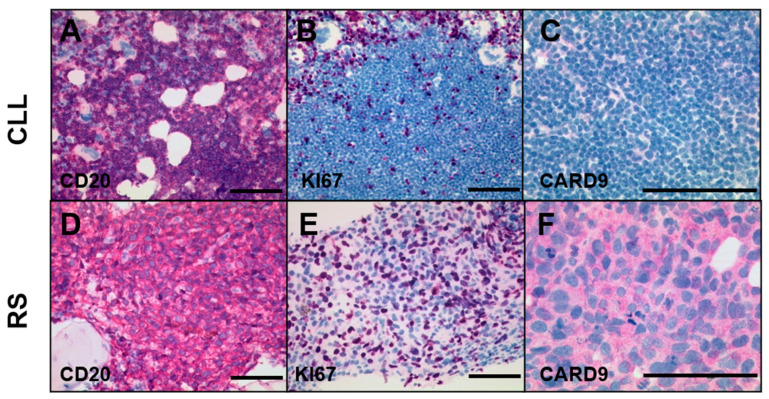
CARD9 staining of one bone marrow specimen with RS and CLL component. (**A**–**C**) CD20 antigen expression, proliferation rate (KI67), and CARD9 staining of the B-CLL component. (**D**–**F**) CD20 staining, KI67 staining, and CARD9 expression of the RS component. Size bars indicate a length of 100 µm.

We analyzed *CARD9* expression in 15 RS tissue samples using immunohistochemical staining. In eight (53%) samples, expression of *CARD9* was detected in the neoplastic CD20-positive B-cell population. A bone marrow aspirate from one patient with RS contained both the aggressive lymphoma and the underlying CLL (Figure 3A–F). The CLL component was represented by an infiltrate of slowly proliferating small lymphocytic cells, which were CD20-positive but negative for CARD9 staining. The aggressive RS component in the same biopsy showed strong CD20 expression, but also cytoplasmic staining for CARD9. Supplementary Figure S2 shows another example of an RS case with *CARD9* expression. In 7 of the 15 RS samples, we were able to study the clonal relationship between the RS and the underlying CLL, while 6 cases were shown to be clonally related RS. The one RS case that was not clonally related to the underlying CLL was among the CARD9-negative cases (Supplementary Table S2). In five of the tested RS samples, we additionally analyzed the corresponding CLL specimen for *CARD9* expression. None of these CLL showed CARD9 positivity, even though three of the corresponding RS did.

We also compared several clinical parameters; i.e., age at CLL diagnosis, DLBCL subtype, and the number of CLL therapy cycles received, but did not find significant differences between CARD9-positive and -negative cases in this small cohort (Supplementary Table S2). 

### 3.4. CARD9 Is Rarely Expressed in De Novo DLBCL, in DLBCL Transformed from Lymphoma Other Than CLL, in cHL, or in CLL 

**Figure 4:**
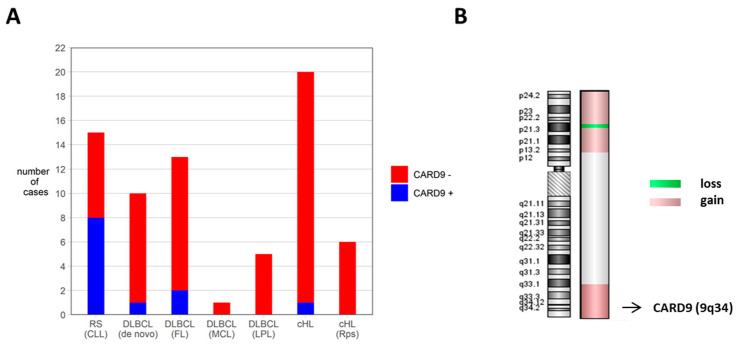
CARD9 expression in RS, non-RS DLBCL, and cHL (**A**) and array CGH of U-RT1, chromosome 9. (**A**) Distribution of CARD9-positive and -negative cases among RS, de novo DLBCL, DLBCL arising from lymphoma other that CLL, and cHL cases using immunohistochemistry. The parent lymphoma type is specified in brackets. (**B**) Array CGH of U-RT1 chromosome 9 including the *CARD9* gene locus (9q34). Abbreviations: FL: follicular lymphoma, cHL: classical Hodgkin lymphoma, MCL: mantle cell lymphoma, LPL: lymphoplasmacytic lymphoma, Rps: relapse.

Using immunohistochemical staining, we found a focal *CARD9* expression in 1 of 10 de novo DLBCLs tested. The de novo DLBCL group included five ABC DLBCLs and five GCB DLBCLs. We also tested 17 DLBCLs that had arisen from lymphomas other than CLL/SLL, and found *CARD9* expression in 2 of these cases. Among the transformed DLBCL, 11 progressed from follicular lymphoma (FL), 5 from lymphoplasmacytic lymphoma, and 1 from a mantle cell lymphoma. In this group, both CARD9-positive cases were DLBCLs that had progressed from a follicular lymphoma. These results indicated that *CARD9* expression is neither a common feature of DLBCL nor a general characteristic associated with the transformation of indolent lymphoma into DLBCL (Figure 4A).

Although most cases of RS are DLBCL, transformation to cHL may occur. We therefore tested 26 cases of cHL, among them 6 lymphocyte-rich and 2 lymphocyte-depleted subtypes, 8 mixed cellularity cHLs, and 10 cases of cHL with nodular sclerosis. Six cases were tumor recurrences. Only a single case of cHL, a mixed cellularity cHL, had CARD9-positive Hodgkin Reed–Sternberg (HRS) cells. We furthermore tested 20 cases of CLL and did not find any *CARD9* expression. 

### 3.5. Array CGH Reveals a Copy Number Gain of CARD9 Gene Locus 

We performed aCGH in our cell line U-RT1 [24] and reanalyzed the data to elucidate the genomic alterations that accounted for high *CARD9* expression. We found a copy number gain of the 9q34 *CARD9* gene locus (Figure 4B), whereas there were no aberrations in gene loci of the other CBM proteins *CARD11* (7p22), *MALT1* (18q21), or *BCL10* (1p22). 

### 3.6. Co-Immunoprecipitation Reveals Assembly of an Alternative CBM Complex Consisting of CARD9, BCL10, and MALT1 in U-RT1

**Figure 5:**
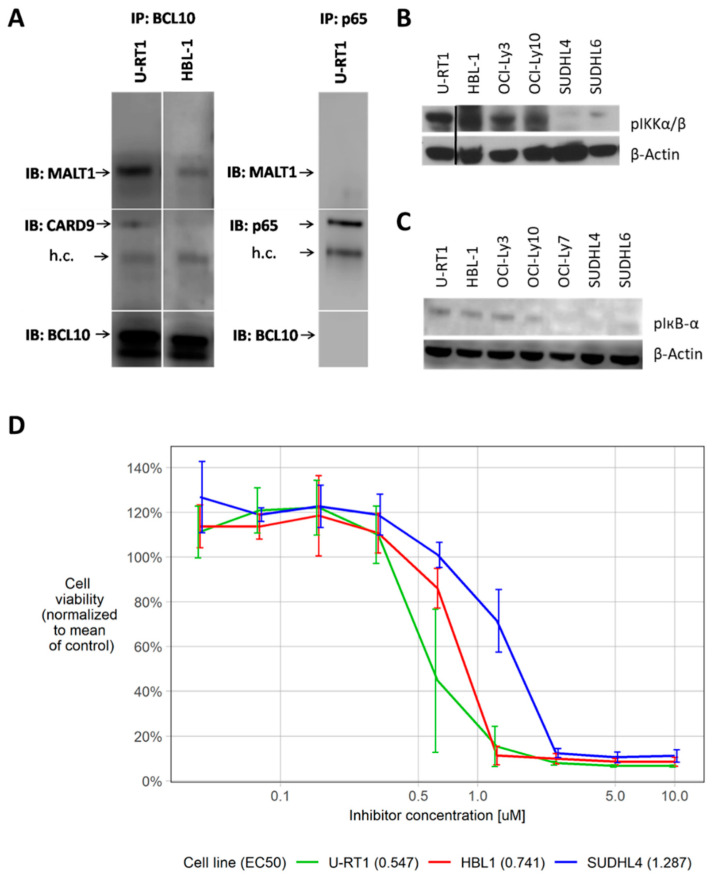
CBM complex formation and NF-кB pathway activation in U-RT1. (**A**) Anti-BCL10 co-immunoprecipitation with immunoblots for MALT1, CARD9, and BCL10 in U-RT1 and HBL-1, as well as anti-p65 purification with immunoblots for MALT1, p65, and BCL10 in U-RT1 as control. (**B**,**C**) Western blot analysis for phosphorylated IκB and IKK in U-RT1; the ABC DLBCL cell lines HBL-1, OCI-Ly3, and OCI-Ly10; and the GCB DLBCL cell lines SUDHL4 and SUDHL6. (**D**) Treatment of U-RT1, HBL-1, and SUDHL4 with NF-кB inhibitor Bay11-7082 for 24 h with EC50 levels. Each inhibition experiment was performed in triplicate. Abbreviations: IP: immunoprecipitation, IB: immunoblot, h.c.: heavy chain.

We speculated that CARD9 might interact with BCL10 and MALT1 to form an alternative CBM complex in U-RT1 cells, and carried out BCL10 co-immunoprecipitation to test this hypothesis. BCL10-containing protein complexes were isolated from cell lysates of U-RT1 and HBL-1 by anti-BCL10 co-immunoprecipitation, and the interaction of BCL10 with CARD9 and MALT1 was determined by immunoblot analysis (Figure 5A). MALT1 was copurified efficiently in both cell lines, but only U-RT1 showed coprecipitation of CARD9. The absence of CARD9 coprecipitation in HBL-1 was consistent with previous results of RT-qPCR analyses, whole-protein Western blots (Figure 1C), and immunostaining of cell precipitates (Figure 2G). Anti-p65 immunoprecipitation and subsequent immunoblotting with anti-BCL10, anti-p65, and anti-MALT1 antibodies revealed no coprecipitation of BCL10 or MALT1 in U-RT1, confirming the specificity of the anti-BCL10 immunoprecipitation. These results indicated the assembly of an alternative CBM complex in U-RT1, consisting of BCL10, MALT1, and CARD9.

### 3.7. Phosphorylated IKK and IкB Indicate NF-кB Activation in U-RT1

Activation of NF-кB generally involves phosphorylation and inactivation of IкB, which is catalyzed by phosphorylated and active IKK. Hence, the presence of phosphorylated IKK and IкB indicate an active canonical NF-кB pathway [28,29]. Immunoblotting revealed phosphorylated IкB in U-RT1, as well as in the ABC DLBCL cell lines HBL-1, OCI-Ly3, and OCI-Ly10, while phosphorylation of IкB was absent in the GCB DLBCL cell lines OCI-Ly7, SUDHL4, and SUDHL6. Immunoblotting for phosphorylated IKK largely showed the same pattern (Figure 5B,C).

### 3.8. Treatment with NF-кB Pathway Inhibitor BAY11-7082 Reduces Viability of U-RT1

Bay11-7082 is a potent inhibitor of the NF-кB pathway, and has been shown to induce apoptosis in a variety of cancer cells [30,31,32]. An MTT assay was performed after treatment with Bay11-7082 to investigate the effect of NF-кB pathway inhibition on U-RT1, HBL-1, and SUDHL4. While all three cell lines tested showed reduced cell viability after 24 h of treatment, U-RT1 cells were the most vulnerable. The GCB DLBCL cell line SUDHL4 showed the lowest sensitivity to NF-кB inhibition. Mean cell viability plotted against increasing inhibitor concentrations and EC50 values are illustrated in Figure 5D. Individual values and confidence limits are given in Supplementary Figure S1.

### 3.9. DNA Sequencing of CARD9, CARD11, MYD88, and CD79A in U-RT1 Exhibits No Activating Mutations 

Activating mutations of *CARD11*, *MYD88*, and *CD79A* are known to be hallmarks of ABC DLBCL, and are often crucial for NF-кB activation in these tumors [21,23]. We performed DNA sequencing to elucidate whether mutations of the aforementioned genes may contribute to the constitutively active NF-кB signaling in U-RT1. Known target sites of *MYD88* (exon 5) and *CD79A* (exon 5 + 6) showed wild-type sequences in U-RT1. For *CARD11*, we performed whole-exon sequencing. None of the common activating mutations was detected. In addition, we conducted whole-exon sequencing of *CARD9* and found no divergence from the wild-type sequence.

### 3.10. CARD9 Knockdown Decreases Cell Viability of U-RT1 

**Figure 6:**
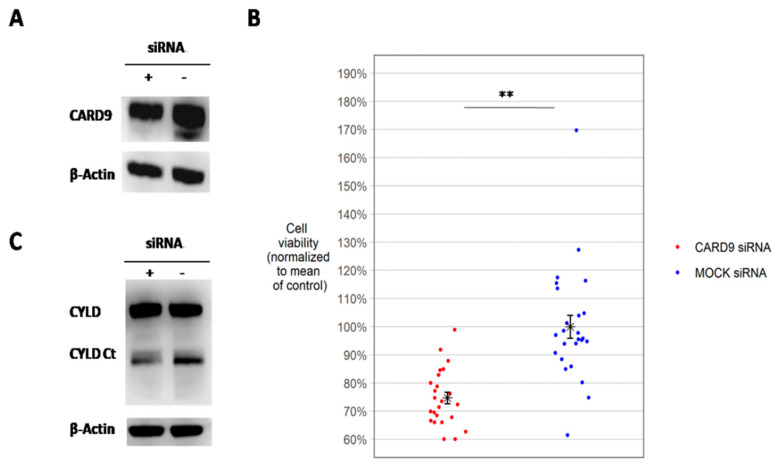
CARD9 knockdown in U-RT1 with cell viability assay and analysis of CYLD cleavage products. (**A**) Western blot of CARD9 protein after siRNA knockdown in U-RT1. (**B**) MTT assay showing cell viability of U-RT1 cells after CARD9 protein downmodulation compared with the control group 24 h after siRNA knockdown using electroporation. Each dot represents one MTT measurement; measurements were obtained from 5 separate electroporation experiments. (**C**) Uncleaved CYLD protein levels and CYLD C-terminal cleavage product after CARD9 knockdown using Western blot analysis. The CYLD Western Blot was repeated four times. ** *p* < 0.01.

The next step was to determine the functional relevance of CARD9 in U-RT1 using siRNA knockdown. We found that even a slight downmodulation of CARD9 protein levels to 72% of control (Figure 6A) led to a significant reduction in cell viability (*p* = 0.0018) (Figure 6B). We also performed MALT1 substrate cylindromatosis (CYLD) Western blot analysis after CARD9 siRNA knockdown, and detected a significant reduction in C-terminal CYLD cleavage product compared with the control group (*p* = 0.017). Loss of CYLD cleavage products indicates reduction in MALT1 paracaspase activity [33].

## 4. Discussion

We demonstrated the expression of *CARD9* in the RS model system U-RT1, as well as in the parent lymphoma of the cell line. CARD9 is known to be an important activator of the NF-кB pathway in myeloid cells [25,27,34,35]. So far, there have been no published data that documented the expression or functional significance of *CARD9* in non-neoplastic lymphoid cells. In the context of neoplasia, CARD9 has been demonstrated in gastric MALT lymphoma. The findings of Nakamura et al. [36] indicated a potential role of *CARD9* overexpression in lymphoma progression and the development of resistance to *Helicobacter pylori* eradication therapy. In addition, gains of chromosomal material, including the chromosome 9q34 gene locus of *CARD9*, have been identified in a subset of gastric MALT lymphomas [37]. In our study, we demonstrated for the first time the expression of *CARD9* in an aggressive B-cell lymphoma.

One possible explanation for the high protein levels of CARD9 in U-RT1 is gene amplification, as already described in MALT-lymphoma [36,37]. Indeed, array CGH analysis revealed copy number gains involving the *CARD9* gene locus in U-RT1. An alternative mechanism to be considered is enhanced gene expression by epigenetic regulation. 

In myeloid cells of the innate immune system, the NF-кB pathway is activated upon ligation of pathogen-associated molecular patterns (PAMPs) or endogenous danger-associated molecular patterns (DAMPs) [38,39] and subsequent stimulation through spleen tyrosine kinase (SYK)-coupled C-type lectin receptors (CLRs) [25,40,41]. In contrast to lymphoid cells, the CBM complex of myeloid cells includes CARD9 instead of CARD11 [25,27,35]. Even though CARD9 and CARD11 are highly homologous in the N-terminal CARD domain and the coiled–coiled domain for protein oligomerization and activation of the CBM signalosome, CARD9 lacks the membrane-associated guanylate kinase (MAGUK) domain, which harbors phosphorylation sites for PKCβ and PKCθ isoforms [34,42,43], and instead is activated by phosphorylation within the coiled–coiled domain at residue T231 by the PKCΔ isoform. CARD9-T231 phosphorylation induces conformational changes that disrupt the autoinhibitory state of CARD9 by exposing its CARD domain for binding of BCL10 [44,45], assembly of the CBM complex, and subsequent NF-кB activation. 

The kinase SYK, which is a common activator of the NF-κB pathway in lymphoid and myeloid cells, was shown to be a linker between CLRs and PKCΔ in myeloid cells [25,34,45]. Interestingly, activation of PKCΔ by SYK was found to be an antiapoptotic driver in chronic lymphocytic leukemia [46,47], and the active protein kinase SYK was also observed in follicular lymphoma [48], DLBCL [49], and mantle cell lymphoma [50]. Together, these findings indicated that PKCΔ, activated by SYK, may be able to phosphorylate CARD9 in RS as well.

Using co-immunoprecipitation, we demonstrated the assembly of a CBM complex with CARD9 instead of CARD11. Western blot analyses of phospho-IKK and phospho-IкB, as well as pharmacological IKK inhibition studies, indicated that the survival of U-RT1 cells depends on NF-кB activation. 

Importantly, knockdown of *CARD9* significantly reduced U-RT1 cell viability and the quantity of CYLD cleavage products as an indicator of impaired MALT paracaspase activity, which is essential for NF-кB pathway activation. These results underlined the relevance of CARD9 in promoting the survival of U-RT1 cells, possibly via NF-кB activation.

To complement our findings, we examined CARD9 protein levels in a series of RS tissue specimens and different lymphoma entities. In particular, we detected *CARD9* expression in the lymphoma cells from 53% of the RS cases, whereas de novo DLBCL and DLBCL arising through transformation of lymphomas other than CLL only rarely exhibited CARD9. Out of 26 cHLs tested, *CARD9* expression was detectable only in the HRS cells of a single case. In addition, 25 CLL cases remained negative for CARD9, even though 5 of the CLL cases later showed transformation into DLBCL. Although the size of our RS cohort was moderate, and further studies with more cases will be required, our results suggested that the expression of *CARD9* is neither a feature of CLL, de novo DLBCL or cHL, nor does it represent a general event in the transformation of indolent lymphoma to aggressive lymphoma. Instead, *CARD9* overexpression as a possible activator of the NF-кB pathway and driver of proliferation appears to be a phenomenon of RS. CARD9 could drive transformation from CLL to RS either autonomously by activating the NF-κB pathway or in combination with other genetic lesions. Chakraborty et al. demonstrated that the combined loss of *TP53* and *CDKN2A* leads to proliferation and transformation of CLL cells in the context of BCR stimulation [18]. Because U-RT1 displays both of these genetic lesions, the question arises whether *CARD9* expression could augment the effect of the underlying cell cycle inhibitor deficiency. Another mechanism of transformation that seems to be independent from *CARD9* expression is activation of AKT via Notch1 [19]. Kohlhaas et al. showed that AKT was activated in high-risk CLL and in 52.6% of RS patients tested. Therefore, AKT activation might account for the transformation of CARD9-negative RS cases of our cohort. Other common lesions that frequently occur in RS, such as MYC and EGR2 activation or Trisomy 12, still need to be elucidated concerning the corresponding signaling pathway and the contribution to the transformational process. 

Until now, most RS patients were treated using a similar therapeutic regimen to that of patients with de novo DLBCL, even though RS and de novo DLBCL have been shown to be distinct disease entities. In view of the poor prognosis of RS patients despite intensive therapy, more targeted approaches to RS are needed. Recently, Leshchiner et al. [51] performed experiments using small-molecule inhibitors that directly targeted CARD9, and registered an attenuation of IKK phosphorylation after CARD9 inhibition in the context of inflammatory bowel disease. In our study, we described the aberrant expression of *CARD9* with oncogenic features in the new RS model system U-RT1, as well as the expression of *CARD9* in a subset of RS cases as a possible pathogenetic mechanism of RS for the first time. As a future prospect, CARD9 inhibition could serve as an approach in the development of novel targeted therapies for RS.

## 5. Conclusions

We have shown the expression of *CARD9* in a subset of RS cases, in the RS model system U-RT1, as well as in the parent lymphoma of the cell line. We demonstrated the assembly of a CBM complex with CARD9 instead of CARD11. Pharmacological NF-κB inhibition and Western blot analyses of phospho-IKK and phospho-IкB indicated constitutive activation of NF-κB in U-RT1. Importantly, knockdown of *CARD9* significantly reduced U-RT1 cell viability and quantity of CYLD cleavage products. These results underlined the relevance of CARD9 in promoting the survival of U-RT1 cells, possibly via NF-кB activation. This is the first report of an ectopic expression of *CARD9* in an aggressive lymphoma. In view of the poor prognosis of RS patients despite intensive therapy, new therapeutic options for RS are needed. As a future prospect, CARD9 could serve as a target for therapeutical approaches in RS. 

## Data Availability

Microarray data are available at GEO under accession number GSE171481. For other original data, please contact julia.maier@uniklinik-ulm.de.

## Supplementary Materials

The following are available online at https://www.mdpi.com/article/10.3390/cancers14030531/s1. Figure S1: Treatment of U-RT1, HBL-1, and SUDHL4 with the NF-кB pathway inhibitor BAY11-7082. Emax fitted regression curves with confidence limits. Table S1: Primer sequences for gene sequencing, quantitative RT-PCR primer sequences, and CARD9 siRNA oligonucleotide sequences. Figure S2: RS case with immunoreactivity for CD20 and CARD9. Table S2: RS cohort with clinical and biological specifications.
