# Supplementary material for: CARD9 Forms an Alternative CBM Complex in Richter Syndrome
Source: Cancers (Basel). 2022 Jan 21;14(3):531. doi: 10.3390/cancers14030531 (PMC8833648; doi:10.3390/cancers14030531)
Supplement: Supplementary file 1 [file cancers-14-00531-s001.zip › cancers-1496609-supplementary.pdf]

cancers

MDPI

Article

CARD9 Forms an Alternative CBM Complex in Richter Syndrome

Julia Maier [1], André Lechel [2], Ralf Marienfeld [1], Thomas F. E. Barth [1], Peter Möller [1,*] and Kevin Mellert [1]

Supplementary Material

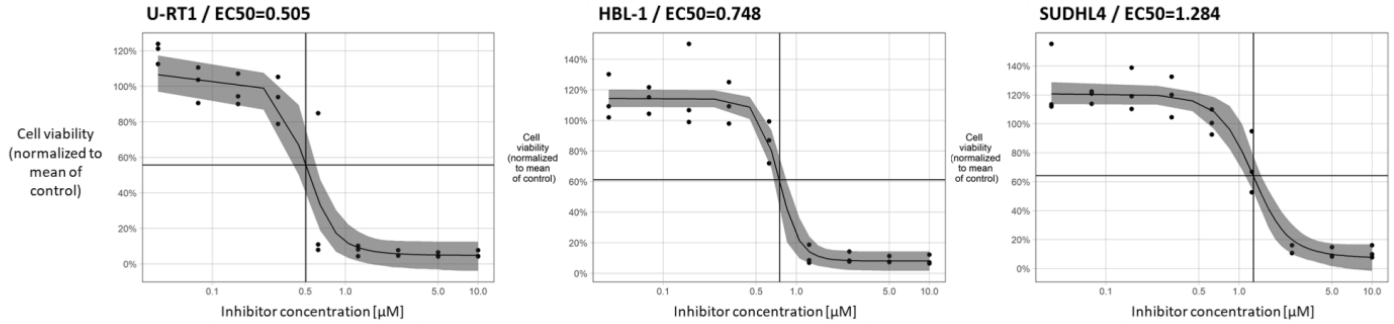

Figure S1. Treatment of U-RT1, HBL-1, and SUDHL4 with the NF-κB pathway inhibitor BAY11-7082. Emax fitted regression curves with confidence limits displayed as grey bands and mean values of technical quintuplicates of each replication as individual dots.

Table S1. Primer sequences for gene sequencing, quantitative RT-PCR Primer sequences and CARD9 siRNA oligonucleotide sequences.

Gene Sequencing Primer	
CD79 Exon 5 forward	5'-GGGCTGGGGGACACTAACACTC-3'
CD79 Exon 5 reverse	5'-TGGGTGCTCACCTACAGACCAC-3'
CD79 Exon 6 forward	5'-TATCTGCTGGTGTGGTTGGG-3'
CD79 Exon 6 reverse	5'-GTCCAGGAAAGGGGTTGGG-3'
MYD88 Exon 5 forward	5'-ACCCCTTGGCTTGCAGGT-3'
MYD88 Exon 5 reverse	5'-AGGCGAGTCCAGAACCAAGATT-3'
**qPCR Primer**	
CARD9 forward	5'-GGAGCTGCAGCAGGAGAA-3'
CARD9 reverse	5'-CTGGATGTAGGGGCTGCTC-3'
CARD11 forward	5'-TACTTCCTGCCCTACCATCC-3'
CARD11 reverse	5'-AAATCTGATGTTCGCTTCAGG-3'
BCL10 forward	5'-AGGTCTGGACACCCTTGTTG-3'
BCL10 reverse	5'-TGGAAAAGGTTCACAACTGCT-3'
MALT1 forward	5'-GGACATCCTTTTGTTCAATATCAGT-3'
MALT1 reverse	5'-TGTTATTAACTCGACAGACATAAAAGC-3'
β-Actin forward	5'-TGTGGCATCCACGAAACTAC-3'
β-Actin reverse	5'-GGAGCAATGATCTTGATCTTCA-3'
**CARD9 siRNA Oligonuleotides**	
Hs_CARD9_8	5'-CTCCAAAGATGACTTCATCAA-3'
Hs_CARD9_7	5'-CTGTTCTGTTTCACCATGTAA-3'
Hs_CARD9_5	5'-ACGTAAGGACTCCAAGATGTA-3
Hs_CARD9_3	5'-CTGGTCATCCGCAAACGGAAA-3'

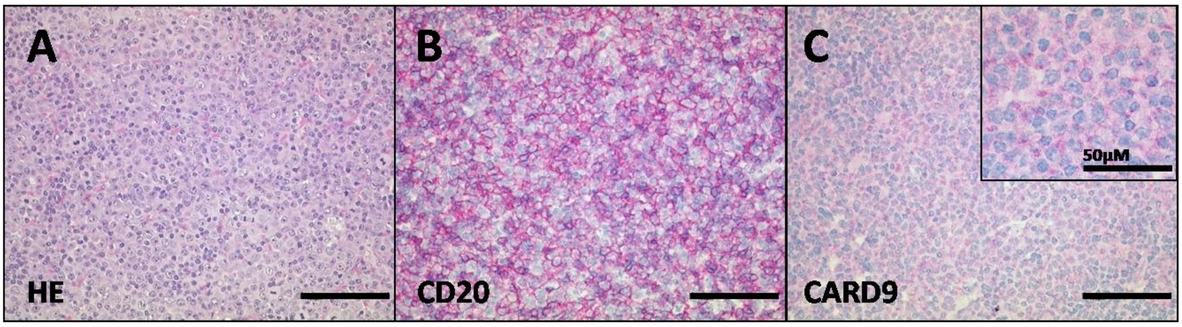

Figure S2. RS case with immunoreactivity for CD20 and CARD9. (A) shows HE staining, in (B) CD20 marks the neoplastic B cell population and in (C) cytoplasmic expression of CARD9 is shown in the majority of lymphoma cells. Size bars indicate a length of 100µm if not otherwise stated.

Table S2. RS cohort with clinical and biological specifications. n.a. = not available. * = censored.

Patient	CARD9	Clonal relationship	Age at CLL diagnosis	Age at RS diagnosis	Survival time after RS	Binet Stadium	Ibrutinib treatment	ABT-199 treatment	Treatment cycles	DLBCL subtype
RS1	positive	n.a.	59	77	17.08*	C	yes	n.a.	n.a.	ABC DLBCL
RS2		n.a.	n.a.	n.a.	20.07*	n.a.	n.a.	n.a.	>1	ABC DLBCL
RS3		yes	58	71	3,96	B	yes	n.a.	n.a.	ABC DLBCL
RS4		n.a.	n.a.	n.a.	n.a.	n.a.	n.a.	n.a.	>1	n.a.
RS5		yes	54	63	1.28	B	no	yes	n.a.	n.a.
RS6		yes	60	66	0.85	B	no	yes	n.a.	ABC DLBCL
RS7		n.a.	n.a.	n.a.	n.a.	n.a.	n.a.	n.a.	>1	n.a.
RS8		n.a.	n.a.	n.a.	n.a.	n.a.	n.a.	n.a.	n.a.	n.a.
RS9	negative	yes	61	65	4.76	B	no	yes	n.a.	GCB DLBCL
RS10		yes	67	73	0.96	n.a.	yes	yes	>1	n.a.
RS11		n.a.	52	58	2.07	n.a.	no	n.a.	n.a.	ABC DLBCL
RS12		n.a.	69	73	2.33	A	no	n.a.	>1	ABC DLBCL
RS13		no	84	84	28.29	n.a.	no	n.a.	>1	n.a.
RS14		n.a.	n.a.	n.a.	n.a.	n.a.	n.a.	n.a.	>1	n.a.
RS15		Yes	63	65	9.07*	C	yes	yes	>1	ABC DLBCL

Western Blot raw data with densitometry using ImageJ

Figure 1C and 5C:

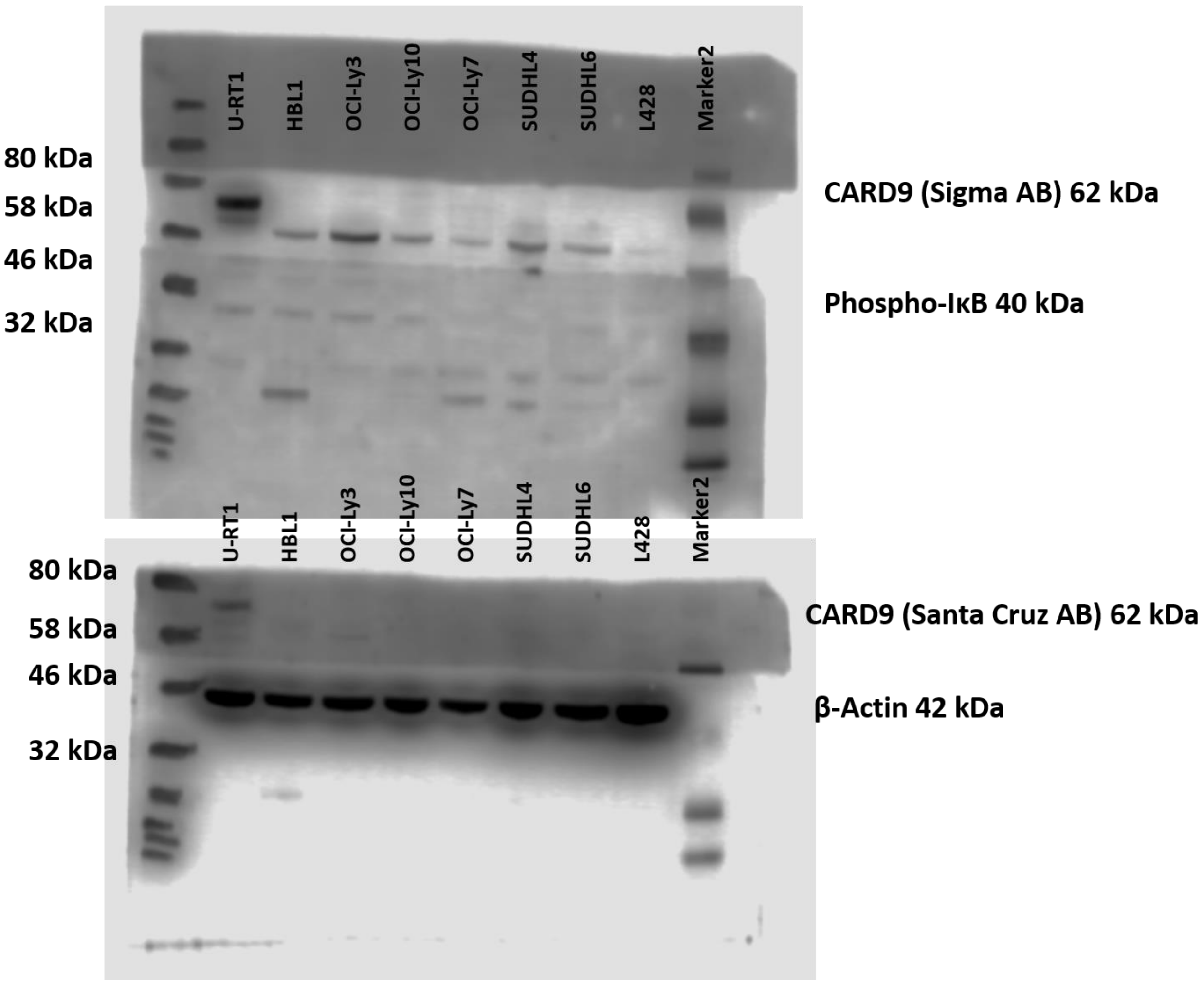

Figure 1C and 5C: Quantification of Western Blot bands using ImageJ

	Card9 (Sigma)	p-IkB	Card9 (Santa Cruz)	beta-Actin
U-RT1	24.981.882	13.947.912	65.983.530	33.745.459
HBL-1	2.993.134	11.780.740	19.250.033	23.950.388
OCI-Ly3	2.071.305	10.476.790	16.931.841	29.079.167
OCI-Ly10	2.746.134	5.496.083	32.610.731	30.434.924
OCI-Ly7	2.672.255	1.596.841	22.176.062	16.621.631
SUDHL4	1.972.477	2.794.740	25.924.811	41.144.945
SUDHL6	3.477.355	2.065.790	22.065.518	49.261.731
L428	4.116.770	2.921.770	16.449.723	63.993.309

Figure 1D

β-Actin = Figure 5B

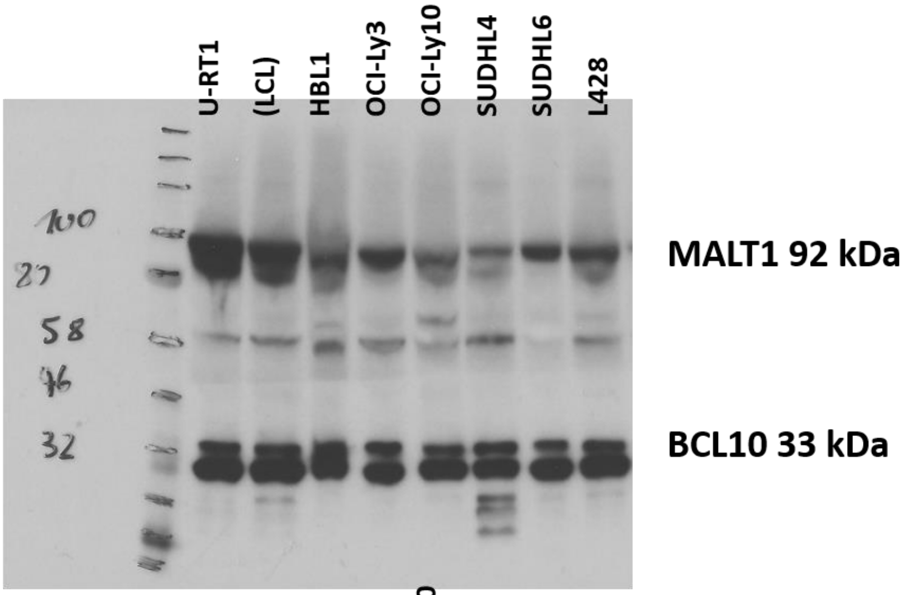

Figure 5B

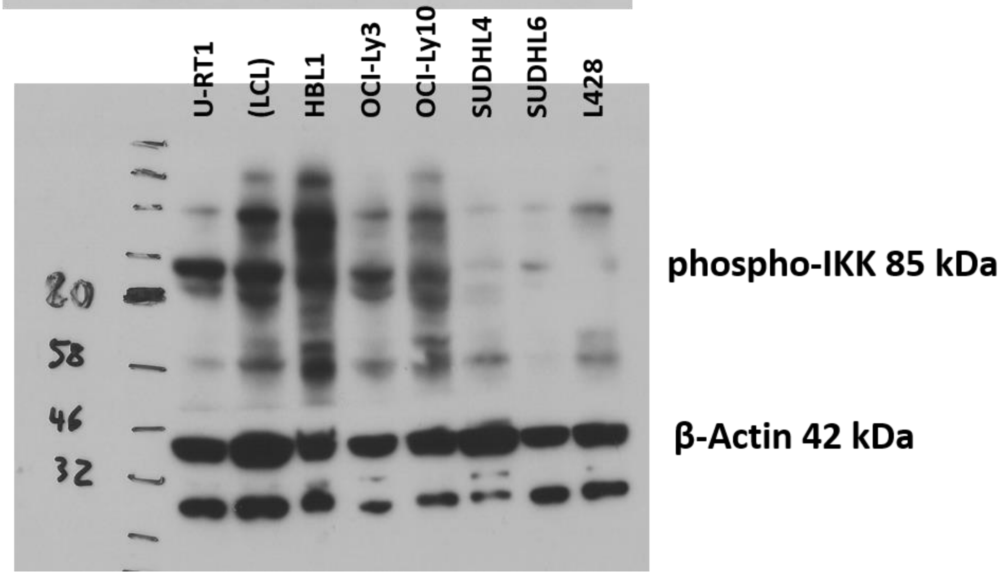

Figure 1D and 5B: Quantification of Western Blot bands using ImageJ				
	**MALT1**	**BCL10**	**p-IKK**	**beta-Actin**
**U-RT1**	91.643.016	80.187.480	73.390.037	69.628.894
**(LCL)**	79.969.844	91.188.258	86.439.187	104.361.501
**HBL-1**	56.266.581	64.271.752	88.498.995	60.142.388
**OCI-Ly3**	46.948.187	66.103.338	55.476.652	54.397.602
**OCI-Ly10**	38.626.995	71.765.238	61.149.258	67.253.359
**SUDHL4**	29.614.530	73.132.480	9.255.045	94.901.087
**SUDHL6**	29.577.217	64.677.995	7.954.439	52.118.924
**L428**	52.911.309	78.268.551	4.224.811	65.424.844

Figure 5A U-RT1 anti-BCL10 IP

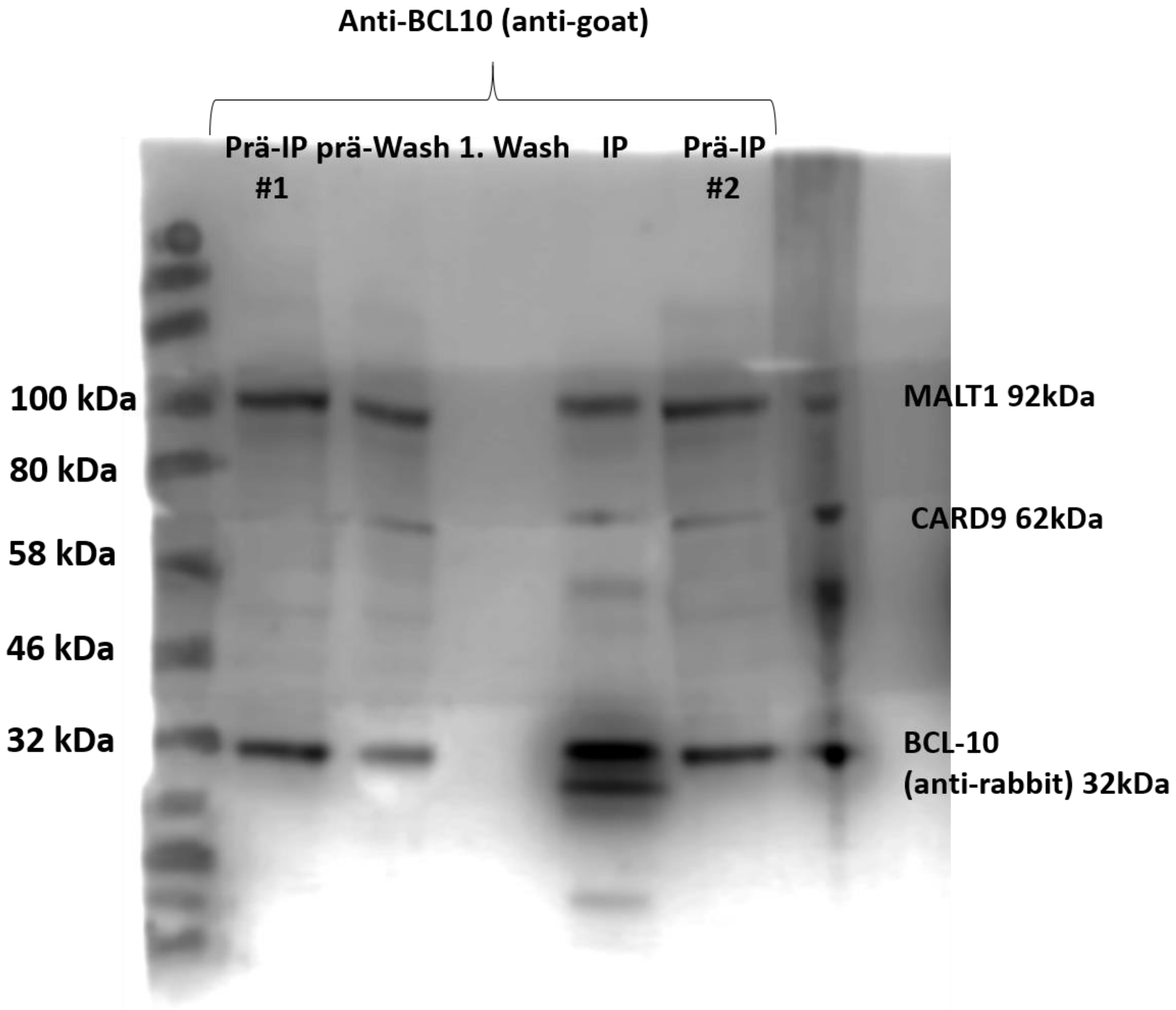

Figure 5A U-RT1 anti-p65 IP

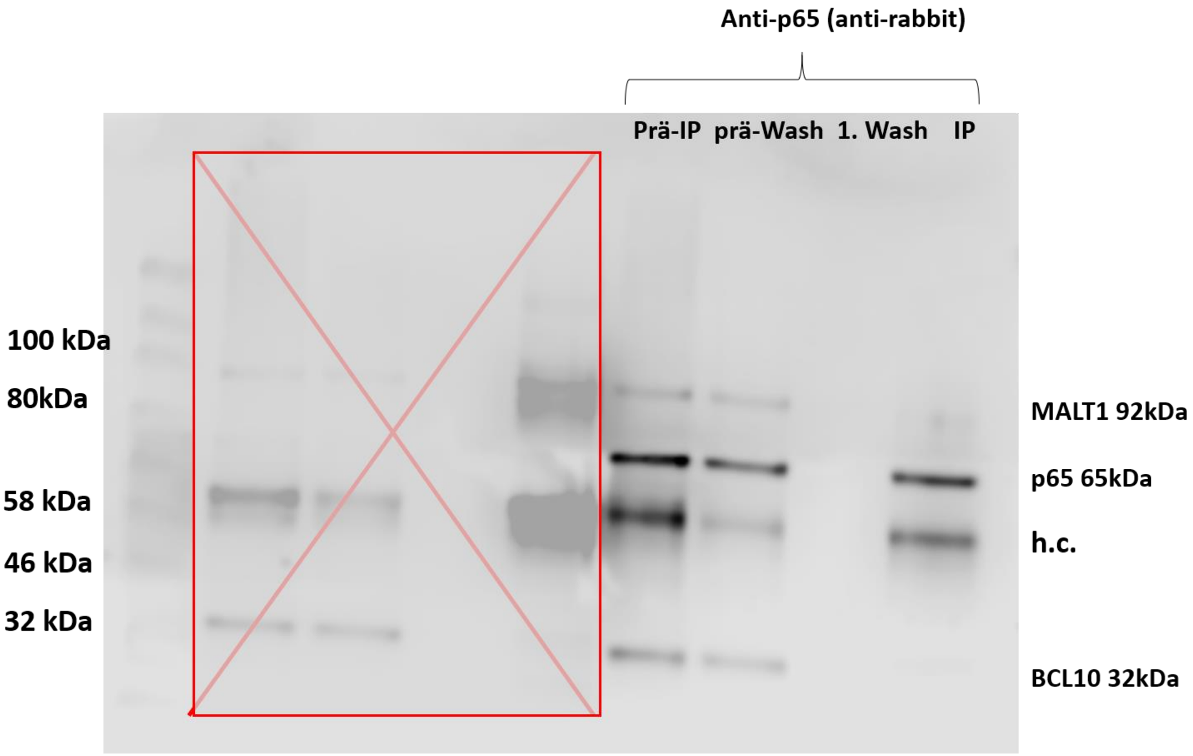

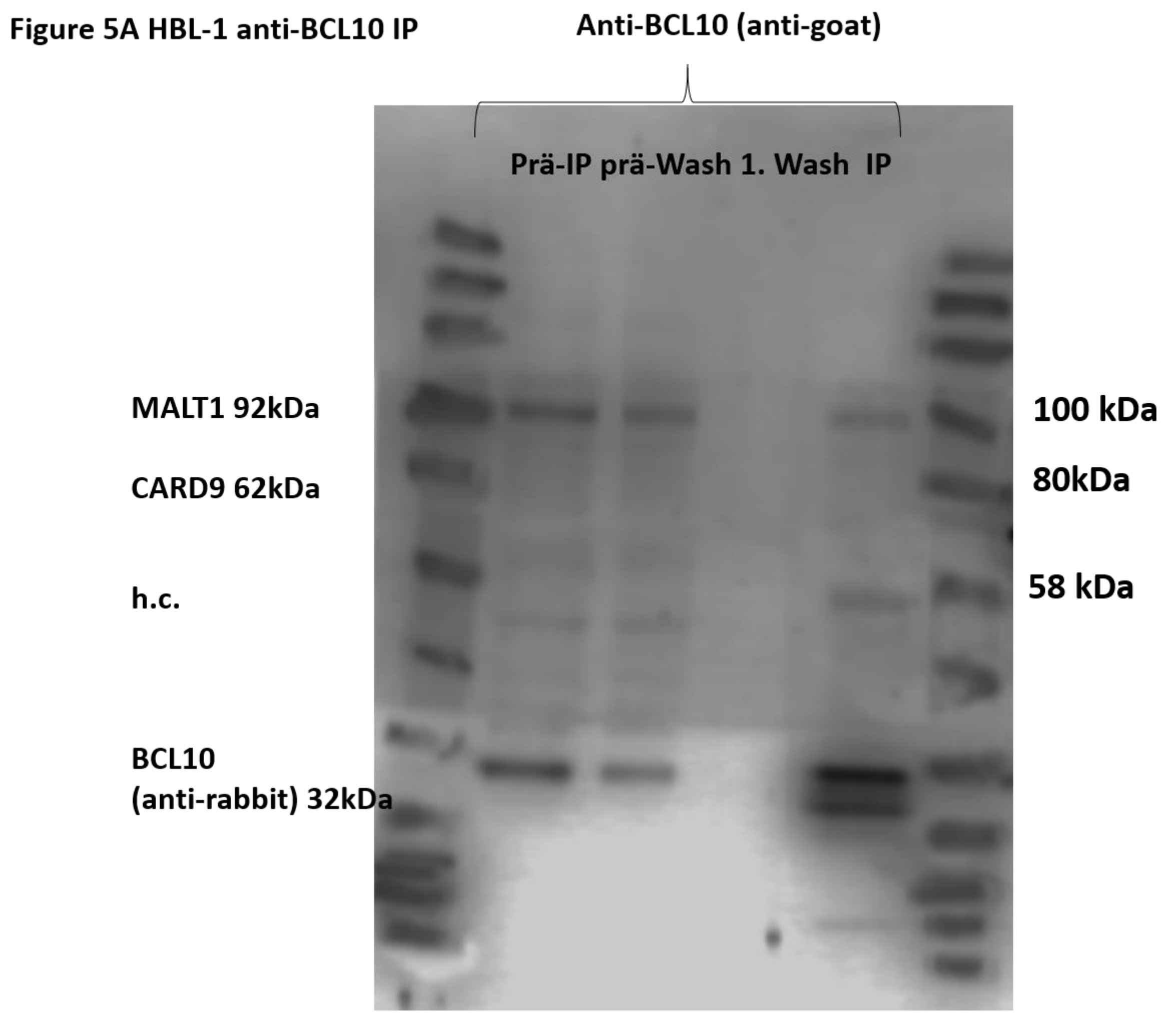
Figure 5A HBL-1 anti-BCL10 IP
Anti-BCL10 (anti-goat)
Prä-IP prä-Wash 1. Wash IP
MALT1 92kDa
CARD9 62kDa
h.c.
BCL10
(anti-rabbit) 32kDa
100 kDa
80kDa
58 kDa

Figure 6A+B: (+) = CARD9 siRNA treatment (-) = MOCK siRNA treatment

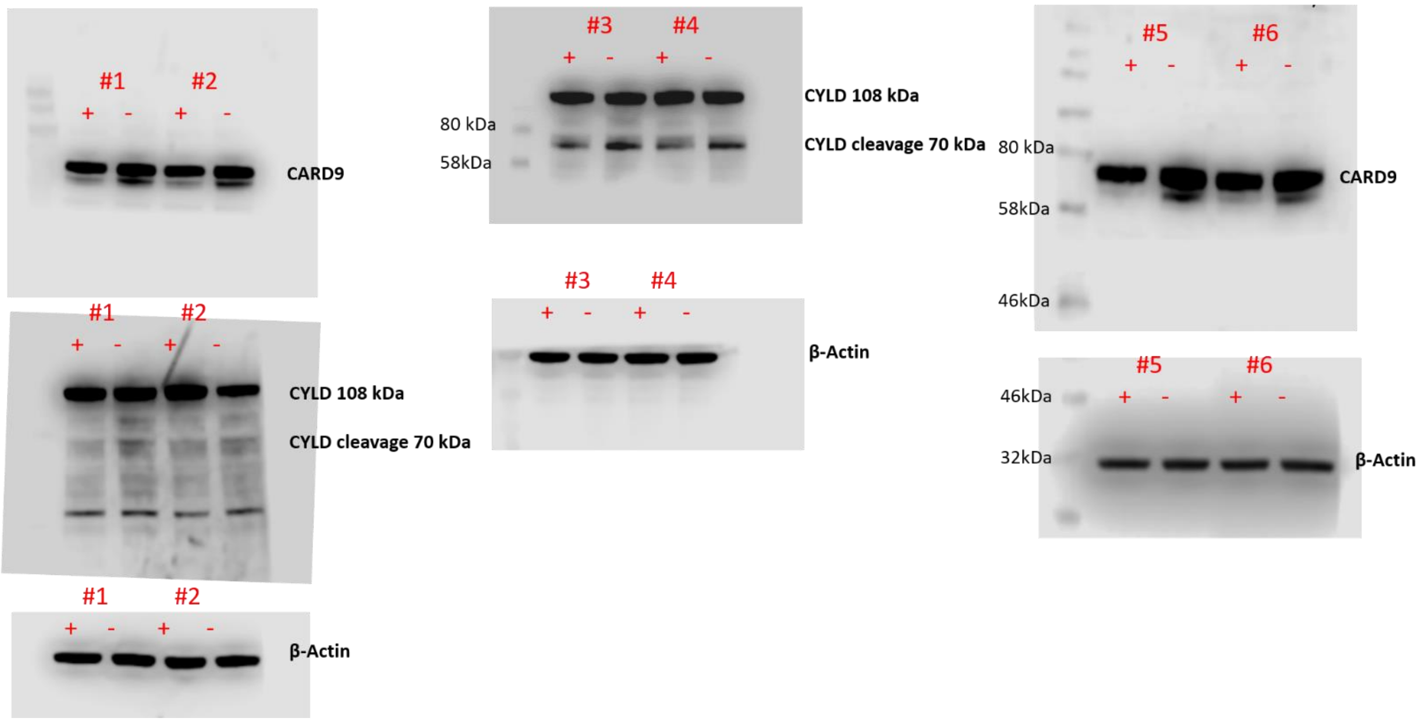

Figure 6A+B: Quantification of Western Blot bands using ImageJ												
**U-RT1**	**#1**		**#2**		**#3**		**#4**		**#5**		**#6**	
**CARD9 siRNA**	+	-	+	-	+	-	+	-	+	-	+	-
**CYLD Ct**	19.306.267	38.536.309	28.909.782	34.209.288	17.749.217	26.651.459	18.761.731	23.521.660				
**beta actin**	32.886.853	35.123.761	32.704.125	31.158.095	22.525.196	25.022.388	30.487.924	27.856.368	19.567.539	20.940.489	20.821.468	23.000.832
**CYLD full**	19.524.125	23.309.418	25.838.953	14.608.882	13.339.953	14.383.175	14.960.175	13.039.104				
**CARD9**	39.108.652	44.642.066	44.643.681	49.062.439					18.216.296	26.407.267	19.726.589	22.618.196
